# Formation of Molybdenum Deuteride at High Pressure: A Neutron Diffraction Study

**DOI:** 10.1021/acs.inorgchem.5c04811

**Published:** 2025-12-18

**Authors:** Zhongsheng Wei, Nicholas P. Funnell, Christopher J. Ridley, Stefan Klotz, Colin R. Pulham, Craig L. Bull

**Affiliations:** † 97008 ISIS Neutron and Muon Facility, Rutherford Appleton Laboratory, Didcot OX11 0QX, U.K.; ‡ Institut de Minéralogie, de Physique des Matériaux et de Cosmochimie, Sorbonne Université, UMR CNRS, Paris 7590, France; § School of Chemistry, University of Edinburgh, David Brewster Road, Edinburgh EH9 3FJ Scotland, U.K.

## Abstract

The locations and occupancies of deuterium atoms in molybdenum deuteride were studied using time-of-flight neutron powder diffraction under pressures up to ∼6.2 GPa. We confirmed the *P*6_3_/*mmc* space group and determined the overstoichiometric deuterium content to give a composition of MoD_1.15_, showing that our data are sensitive to deuterium positions and occupancies. In MoD_1.15_, the majority of the interstitial deuterium atoms occupy the octahedral sites, and the remainder occupy the tetrahedral sites and exhibit relatively short interatomic distances of 1.49 and 1.90 Å to molybdenum atoms.

## Graphical abstract

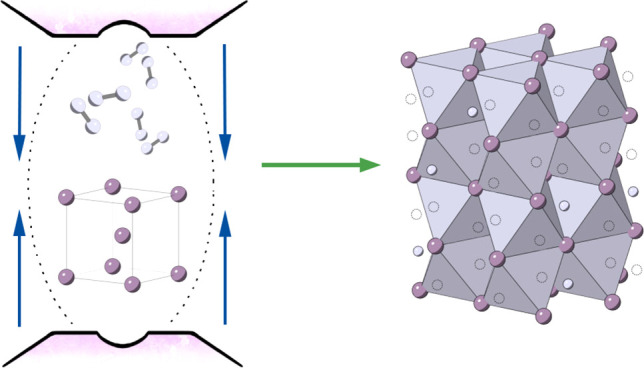

## Introduction

The hydrogen economy offers a sustainable pathway to meet global energy demands while reducing carbon emissions. One of the key challenges in transitioning to hydrogen-based energy systems is efficient hydrogen storage. Current methods such as compressed gaseous storage (CGH_2_) and liquid hydrogen storage have significant limitations, such as high energy demands for compression and liquefaction.^1^ Metal hydrides offer promising solutions due to their significantly higher volumetric energy densities. For example, MgH_2_ has a volumetric energy density of 3.67 kWh/dm^3^,^2^ which is around 4 times larger than that of CGH_2_ at 0.8 kWh/dm^3^ at 350 bar.^3^ Beyond energy density, metal hydrides also provide additional advantages, such as reduced explosion risks, repeatable usage, and no hydrogen losses.^1^ These characteristics make metal hydrides an appealing choice for various applications, requiring mid- to long-term hydrogen storage.

Nearly all transition metals in groups VI–X can form hydrides, making them potential candidates for efficient hydrogen storage materials. Most of these metals have been observed to form hydrides with a H:metal ratio of approximately 1:1, where metal atoms are arranged in face-centered cubic (fcc), hexagonal close-packed (hcp), or double hcp sublattices, while hydrogen atoms occupy octahedral interstitial sites.^4^ There are multiple routes to synthesize metal hydrides, such as high-energy ball milling, hydriding chemical vapor deposition, hydrogen plasma reaction, electrochemical deposition, and melt spinning.^1,5^ For metals that can easily form hydrides, the most common method is direct interaction of metals with pressurized H_2_ gas. This process is achievable as the chemical potential of hydrogen increases steeply with pressures greater than 1 GPa,^6^ providing enough energy for chemical reactions to occur. Examples include the synthesis of ReH_0.85_,^7,8^ PtH,^9^ RuH,^10^ and NiH.^11,12^

Recently, Kuzovnikov *et al*. synthesized a molybdenum hydride with H/Mo = 1.35 using a diamond anvil cell (DAC).^13^ Molybdenum hydride was first discovered in 1977 by the exposure of molybdenum metal to a high hydrogen pressure of 4 GPa^14^ and was later found to be metallic and superconductive.^15^ This result is notable as it demonstrates that molybdenum possesses hydrogen:metal ratios exceeding 1, a rare property in transition metal hydrides at such low pressure. At higher pressures, more hydrogen-rich hydrides have been reported, such as WH_1.3_,^16^ Ni_2_H_3_,^17,18^ Ru_3_H_8_,^19^ Cr_2_H_3_, and CrH_2_.^20^ However, some of these are challenging to access, as the synthesis pressures exceed 50 GPa. In ref 13, Mo metal—a body-centered cubic (bcc) structure—was found to transition to a hcp structure at room temperature and 4.2 GPa, resulting in a hydride with H/Mo = 1.1. This value is indirectly measured from volumetric consideration and is consistent with previous studies showing H/Mo values around 1–1.1.^21–23^ However, unlike previous studies that determined a stable H/Mo ratio of 1–1.1, the authors observed a further increase in MoH_*x*_ cell volume with increasing pressure up to 15 GPa and reported a hydrogen-saturated Mo hydride with a nonstoichiometric value of 1.35.

Although multiple groups^13,23^ have reported Mo hydrides with H/Mo >1, the atomic connectivity of the overstoichiometric H atoms remains uncertain. The most widely accepted structure is that interstitial tetrahedral sites are partially occupied in addition to fully occupied octahedral positions.^4,22–25^ Though an early neutron powder diffraction study on MoH_1.19_ ^26^ (with the H/Mo ratio determined via volumetric analysis) suggested that tetrahedral sites were likely unoccupied while octahedral sites were 95(5)% occupied, the authors also ruled out vacancies in the Mo sublattice as a possible reason for the overstoichiometric hydrogen content. In contrast, a recent neutron powder diffraction study^24^ observed a 4(1)% occupancy of D on tetrahedral sites in Mo­(D + H)_1.07_ (though the (D + H)/Mo ratio was determined via a hot extraction method), with octahedral sites only 93% occupied by D atoms. The determination of H/D site occupancies via diffraction has thus far not been demonstrated conclusively.

In this work, we present the in situ synthesis of MoD_1.15_, observing its formation by neutron powder diffraction. We chose to use D instead of H to avoid incoherent scattering from the latter. Neutron scattering is particularly good for studying metal deuterides as the technique is highly sensitive to light elements such as D, allowing for precise structural analysis even in the presence of heavier elements like Mo. Pressure is achieved with a Paris-Edinburgh (PE) press using a sample volume of 30 mm^3^, which is significantly larger than that of previous DAC experiments (less than 0.02 mm^3^). This larger sample volume provides a more representative average of the structure and offers insights into the practicalities of similar reactions for potential industrial applications. Using this approach, we were able to unambiguously determine the D:Mo ratio as well as the locations of the nonstoichiometric D atoms.

## Experimental Section

Finely ground Mo powder was loaded into a gas clamp with D_2_ gas at 2 kbar, applying a well-established offline high-pressure gas loading technique, as described in ref 27. The sample was contained in a copper–beryllium alloy toroidal gasket, which does not become embrittled in the presence of high-pressure D_2_. The gas clamp, equipped with zirconia-toughened alumina (ZTA) anvils, sealed the 2 kbar gas pressure before its use in the PE press. The D_2_ gas within the gasket also acted as the pressure-transmitting medium (PTM), while the Mo powder acted as a pressure marker using a known equation of state.^28^

We performed the time-of-flight neutron powder diffraction measurements using a VX3 PE press on the PEARL instrument at the ISIS Neutron and Muon Source.^29^ During the experiment, the PE press transmitted a hydraulic load via an oil-driven piston, and the applied load on the press was progressively increased, ultimately reaching the upper limit pressure of ZTA anvils (6–7 GPa). **Caution!** Deuterium is classified as a GHS Flammable Gas, Category 1. Data processing involved applying an initial attenuation correction using Mantid,^30^ followed by Rietveld refinement with Topas Academic V6.^31^ Details of the refinement procedure and fits to data are provided in the Supporting Information.

## Results and Discussion

The neutron powder diffraction patterns of the Mo–D_2_ system as a function of pressure[Fn fn1] are shown in Figure 1. The patterns contain contaminant reflections, from the ZTA anvils and gasket, in addition to those from the sample. No sample peak broadening is observed, indicating that the pressure environment remained hydrostatic due to the pressure-transmitting effect of D_2_. For pressures up to 3.9 GPa, the patterns exhibit no significant changes, suggesting that no phase transition or chemical reaction occurred at these pressures. From 4.4 GPa onward, changes in the diffraction pattern are observed, including the appearance of a new diffraction peak at 1.45 Å and a subtle increase in peak intensity at 2.51 Å, as highlighted in the dashed boxes in Figure 1. Meanwhile, the diffraction peak at 2.21 Å undergoes a significant decrease in intensity and exhibits slight broadening at higher pressures, as marked by the second dashed box. Since Mo remains in the same phase up to 94 GPa and 3470 K,^28^ the observed changes in the diffraction pattern can be attributed to the formation of a new compound. Given its previous report in ref 13, we believe that MoD_*x*_ was successfully synthesized through direct interaction with D_2_ gas, starting at some point between 3.9 and 4.4 GPa. The slight broadening arises from the overlap between the diffraction peaks of MoD_*x*_ and Mo, while the significant intensity drop is likely due to a reduction in the concentration of pure Mo metal within the illuminated gasket volume, suggesting that the formation of MoD_*x*_ continued as the pressure increased further (see Supporting Information).

**Figure 1:**
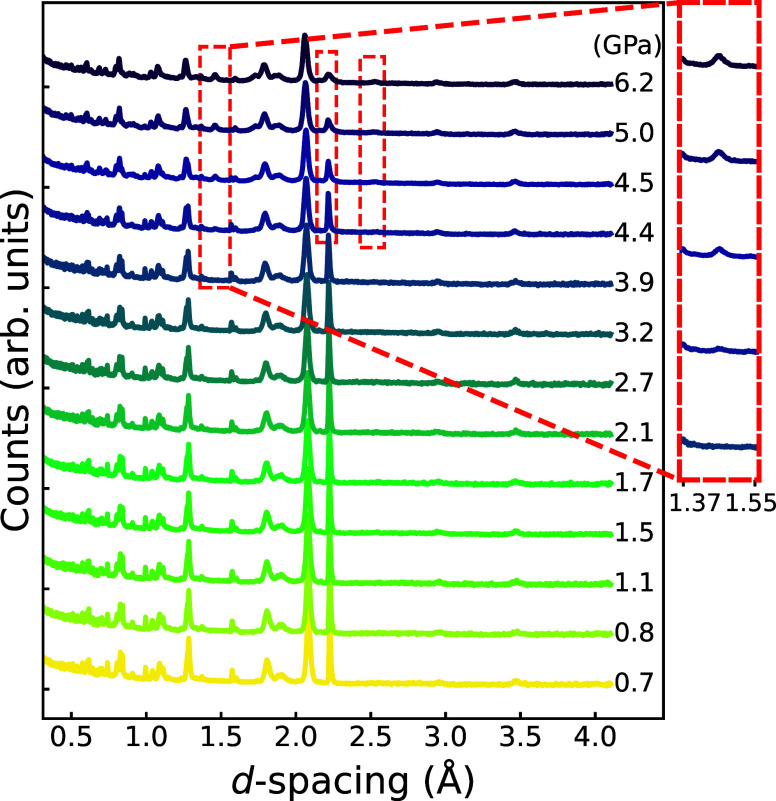
Measured neutron diffraction patterns of Mo in D_2_ gas with increasing pressure. The values to the right of the curves indicate the corresponding pressures, determined by fitting the previously reported equation of state of Mo.^28^ The dashed boxes in the main plot are guides for key changes in the diffraction patterns during the synthesis of MoD_*x*_, while one of these is magnified in the inset on the right.

### Symmetry and Stoichiometry Determination

As discussed previously, MoD_*x*_ (1 < *x* < 2) can adopt a hcp structure with fully occupied octahedral sites and partially occupied tetrahedral sites.^4,22,23,25^ This assumption is based on the fact that the *P*6_3_/*mmc* space group should be adopted by MoD when D/Mo ≈ 1.^22^ In this structure, as illustrated in Figure 2, each Mo atom (Wyckoff site 2*c*) is associated with one octahedral site (2*a*) and two tetrahedral sites (4*f*). Hereafter, D atoms occupying octahedral and tetrahedral sites will be referred to as D_O_ and D_T_, respectively. The D_O_ atoms are located at high-symmetry positions along the edge of the unit cell, while the D_T_ atoms share the same *x*- and *y*-coordinates as the Mo atoms. Another possible model, which has also been suggested as the most probable structure for MoD_2_,^25^ is a similar structure but with a lower-symmetry space group, *P*6_3_ *mc*. An analogous symmetry reduction has been observed previously in a neutron scattering study of TaH_2.2_.^32^ A key characteristic of this symmetry reduction is that the two tetrahedral sites in the *P*6_3_/*mmc* structure become crystallographically inequivalent, in this case, leading to the occupation of a single tetrahedral site in the *P*6_3_ *mc* structure. In order to assess the sensitivity of our neutron data to atomic sites and occupancies, and the ability to discriminate between these models, we analyzed the main reflections through a series of simulated neutron diffraction patterns.

**Figure 2:**
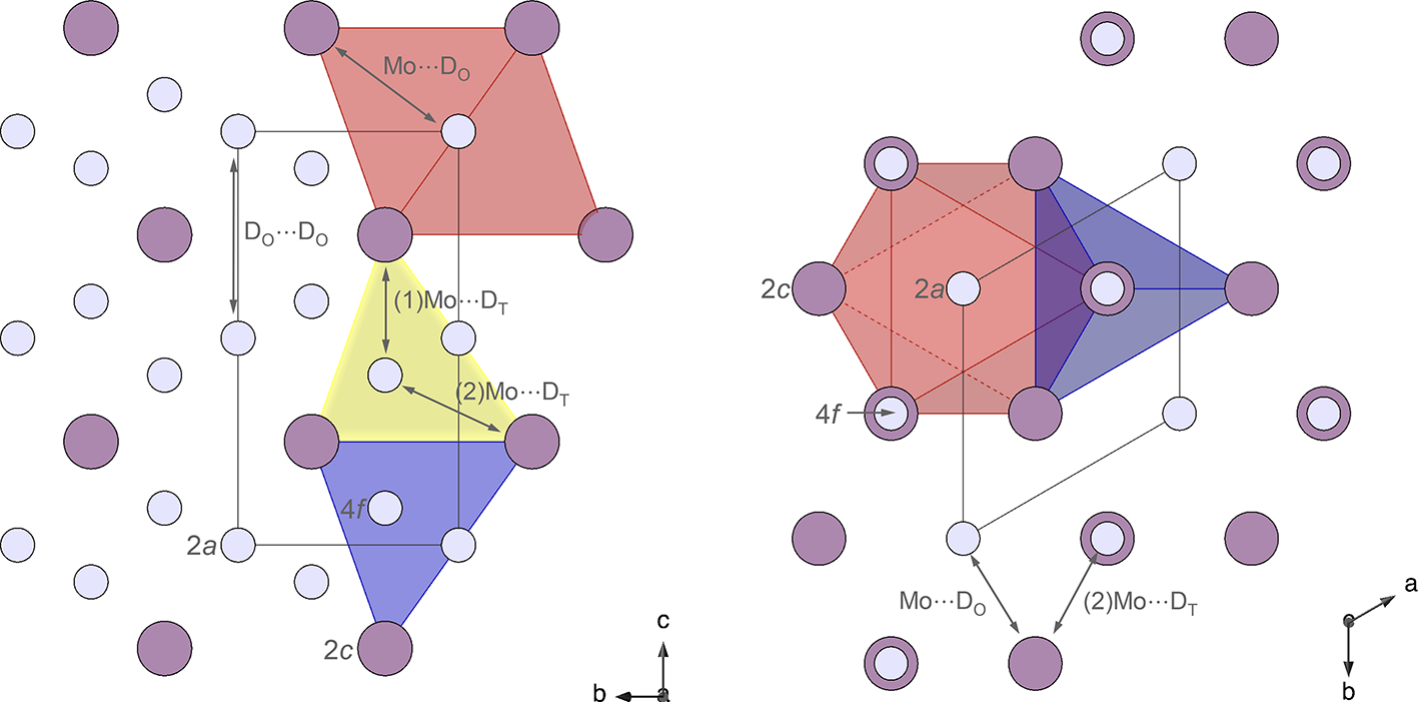
Crystal structure of MoD_*x*_ with the space group *P*6_3_/*mmc*. Purple spheres represent Mo atoms, while gray spheres represent D atoms. Atomic distances are indicated by arrows, and the relevant Wyckoff sites are indicated. One octahedron is highlighted in pink, and two tetrahedra are highlighted in yellow and blue. Some atoms are omitted for clarity; the full contents are shown in each unit cell (black lines).

First, we simulated neutron diffraction patterns of MoD_1+δ_ (0 ≤ δ ≤ 1), where δ represents the number of D_T_ atoms per Mo atom, for both the *P*6_3_ *mc* and *P*6_3_/*mmc* space groups. Based on previous studies,^13,26^ the octahedral sites were reasonably assumed to be fully occupied. The variations in the calculated relative intensities (*I*/*I*_0_, where *I*_0_ is the intensity at δ = 0) of the six main reflections as a function of the number of D_T_ per Mo are illustrated in Figure 3b. For both structures, most reflections increase in intensity with an increasing D_T_ content. The simulated neutron diffraction patterns of both structures with δ = 1 are shown in Figure 3a for comparison. It can be observed in both panels that only two reflections exhibit insensitivity to the structural difference, while all other reflections show differing degrees of sensitivity to the presence of preferential occupation in the tetrahedral sites.

**Figure 3:**
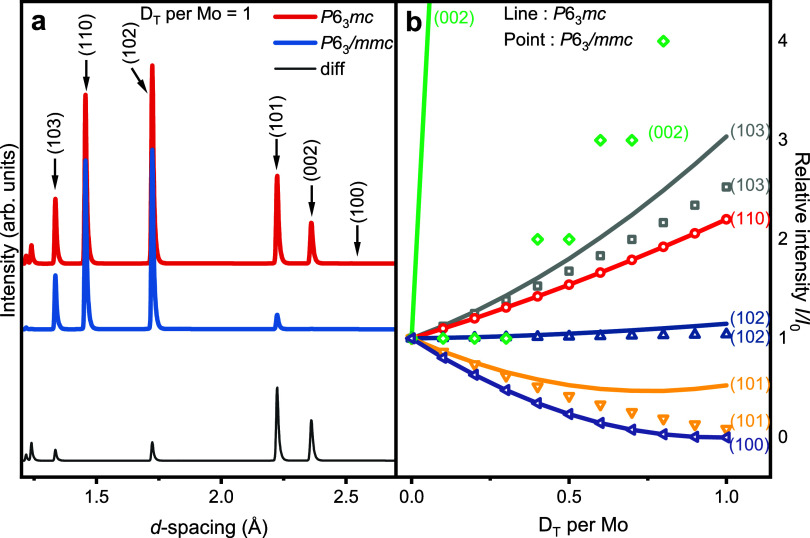
Simulation results of MoD_1+δ_ (0 ≤ δ ≤ 1), where δ represents the number of D_T_ atoms per Mo atom (with D_O_ sites fully occupied). (a) Simulated diffraction patterns at δ = 1. The red pattern corresponds to the *P*6_3_ *mc* structure, while the blue pattern corresponds to the *P*6_3_/*mmc* structure. The gray line represents the difference between the two patterns. (b) Variation in the relative intensities of typical reflections as a function of δ. Lines represent the data for the *P*6_3_ *mc* structure, while points represent the data for the *P*6_3_/*mmc* structure. The apparently erroneous behavior shown by the (002) reflection is a consequence of differing rates of change between the two models and limitations on precision by the refinement software.

With the reduction in symmetry to *P*6_3_ *mc*, the octahedral site is no longer constrained by symmetry to be positioned at the center of the octahedron. Thus, we displaced the D_O_ atom by −0.117, as suggested by ref 25, and carried out another simulation (see Table S1 and Figure S1 in the Supporting Information). We found that after the displacement, the (101) reflection is stronger than (102), and the (002) reflection becomes one of the most intense, while the opposite is observed when D_O_ atoms sit at the center of the octahedron. These further indicate that the neutron diffraction patterns are sensitive to the structural difference. Moreover, as a previous study has suggested that the octahedral site may not be fully occupied,^26^ we also carried out simulations with varying D_O_ and D_T_ content. The results show that the intensities of the (103) and (101) reflections vary only with D_T_ content, whereas the remaining four reflections are sensitive to both D_T_ and D_O_ content to different extents. In conclusion, the neutron diffraction patterns of MoD_*x*_ are sensitive enough to structural variations and occupancy changes, and our data are capable of providing precise structural information for MoD_*x*_. Additional details on the simulation parameters, including lattice constants, atomic positions, and occupancies as well as selected results, can be found in Supporting Information.

The number of contaminant phases in our experiment setup makes refinement challenging, and we found that several parameters became unstable during free refinement. We identified the most favorable values of the octahedral and tetrahedral site occupancies, as well as the tetrahedral site *z*-coordinate, by holding these parameters fixed and incrementally varying each of these, building a multidimensional heat map of *R*_wp_ values. The values we quote here are identified from a clearly localized minimum—full details are provided in the Supporting Information.

We first tested whether the crystal structure of MoD_*x*_ required a reduction in symmetry to *P*6_3_ *mc*. The atomic parameters resulting from this space group setting at 6.2 GPa—where MoD content is greatest—are presented in Table 1. The *z*-coordinate of D_O_ refined to near zero from the initial value of −0.117, suggesting that the D_O_ atoms do not need to deviate from their high-symmetry positions. More importantly, as reducing the symmetry does not lead to a significant improvement in fit quality (see Figure S3), the most appropriate crystal structure is the higher-symmetry structure with the space group *P*6_3_/*mmc*.

**Table 1:** Refined Atomic Coordinates and Occupancies of MoD_*x*_ at 6.2 GPa in *P*6_3_ *mc* and *P*6_3_/*mmc* ^a^

space group	atom label	Wyckoff site	*x*	*y*	*z*	Occupancy
*P*6_3_ *mc*	Mo	2*b*	1/3	2/3	1/4	1
	D_O_	2*a*	0	0	0.007(6)	0.92
	D_T_	2*b*	1/3	2/3	0.565	0.29
*P*6_3_/*mmc*	Mo	2*c*	1/3	2/3	1/4	1
	D_O_	2*a*	0	0	0	0.88
	D_T_	4*f*	1/3	2/3	0.565	0.135

a Where errors are not shown, this is due to the refinement approach. See Supporting Information for details. Note that the occupancy of 0.29 in the *P*6_3_ *mc* model is nearly equivalent to 0.135 in the *P*6_3_/*mmc* model due to the symmetry.

The refined lattice parameters and unit-cell volumes for the *P*6_3_/*mmc* structure are presented in Table 2. An example of the refined diffraction patterns is shown in Figure 4, and the corresponding atomic parameters are listed in Table 1. Our lattice parameters and unit-cell volumes are in good agreement with previously published values,^23,24^ with slight discrepancies arising from differences in the experimental environment. The *c*/*a* ratios of the lattice parameters are approximately 1.62 across all pressure points, which are also in good agreement with previous reports of ∼1.6.^13,23^ At 6.2 GPa, the *z*-coordinate for D_T_ was determined as 0.565. The occupancy of D_O_ was determined as 0.880 and the occupancy of D_T_ was determined as 0.135. This demonstrates that D atoms preferentially occupy the octahedral sites, as expected, but do not completely reach full occupancy. Such incomplete occupancy of the octahedral sites has also been reported previously.^26^ The occupancies of two tetrahedral sites and one octahedral site combine to a nonstoichiometric composition of MoD_1.15_. In the literature, the stoichiometry of metal hydrides is often derived from analysis of the unit-cell volume; applying this method to our current work gives a stoichiometry of MoD_1.11_.^33^ In this study, we have determined D content from diffraction data. The stoichiometries obtained are in good agreement and likely fall within error of each other given sensitivity limitations in the approaches used.

**Table 2:** Lattice Parameters and Unit-Cell Volumes of the Synthesized MoD, Refined Using the Space Group *P*6_3_/*mmc*, with Reference Values for Comparison

pressure (GPa)	*a* (Å)	c (Å)	volume (Å^3^)
4.35(3)	2.918(3)	4.752(10)	35.04(10)
4.50(5)	2.9126(11)	4.724(4)	34.71(4)
5.04(12)	2.9097(9)	4.722(3)	34.63(3)
6.17^a^	2.9092(10)	4.723(3)	34.62(3)
Ambient^23^ ^b^	2.923(2)	4.741(3)	35.08(5)
Ambient^24^ ^c^	2.931(4)	4.745(6)	35.30(11)

a Error is unavailable as it was inferred from the load-pressure relation. There is no detectable quantity of Mo for reliable pressure determination at this pressure point. Note that the uncertainties on all other pressure points are from error propagation in the Rietveld refinements, derived from the Mo equation of state.^28^

b Reference X-ray diffraction data measured at 85 K and 1 atm for MoH_1.11(3)_ quenched from 3.9 GPa and 773 K, with D_O_ 0.95(5)% occupied.

c Reference neutron diffraction data measured at 100 K and 1 atm for Mo­(D + H)_1.07_ quenched from 7.4 GPa and 573 K.

**Figure 4:**
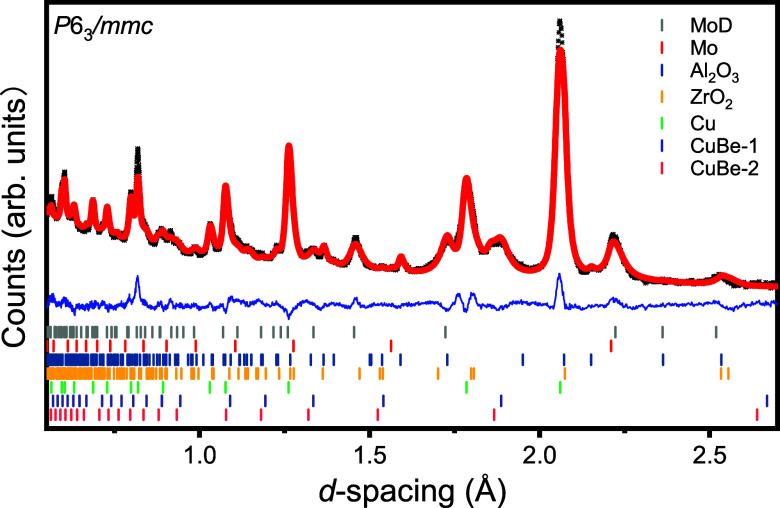
Neutron powder diffraction patterns of MoD_*x*_ with the space group *P*6_3_/*mmc* at 6.2 GPa. The Rietveld fit is represented as a red line, while the experimental data are represented by black marks. The residual is shown as a blue line. Vertical tickmarks indicate the positions of reflections from MoD_*x*_, Mo, anvils (Al_2_O_3_ and ZrO_2_), and gasket (Cu and CuBe), respectively. Note that the two CuBe components correspond to the same impurity phase at two different scattering locations.

### Crystal Structure Analysis

Figure 5 shows unit-cell volumes as a function of pressure. For pressures below 3.9 GPa, there is not yet a clear signature of any reaction between Mo and D_2_; thus, the Mo remains in its bulk metallic form with the space group I*m*3̅*m*. For pressures above 4.4 GPa, where new diffraction peaks appear, the formation of MoD_1.15_ is initiated. The relatively large error bar at 4.4 GPa arises from the fact that only a small amount of MoD_1.15_ has formed at this pressure, increasing the uncertainty in the refinement. Unlike the results for MoH_1.35_,^13^ where a continuous increase in MoH_*x*_ composition from 4 to 15 GPa was inferred from the hcp MoH_*x*_ unit-cell volume increase, we do not observe any volume increase at pressures above 4.4 GPa. We therefore consider the D content to remain unchanged across the four pressure points and use the occupancies determined at 6.2 GPa to be representative of these. It should be noted that as the amount of the D_2_ starting material was not measurable, and the Mo content fell below detectable limits at the highest pressure, we cannot establish this as the saturation limit of D content in MoD_*x*_: a higher D content may be possible.

**Figure 5:**
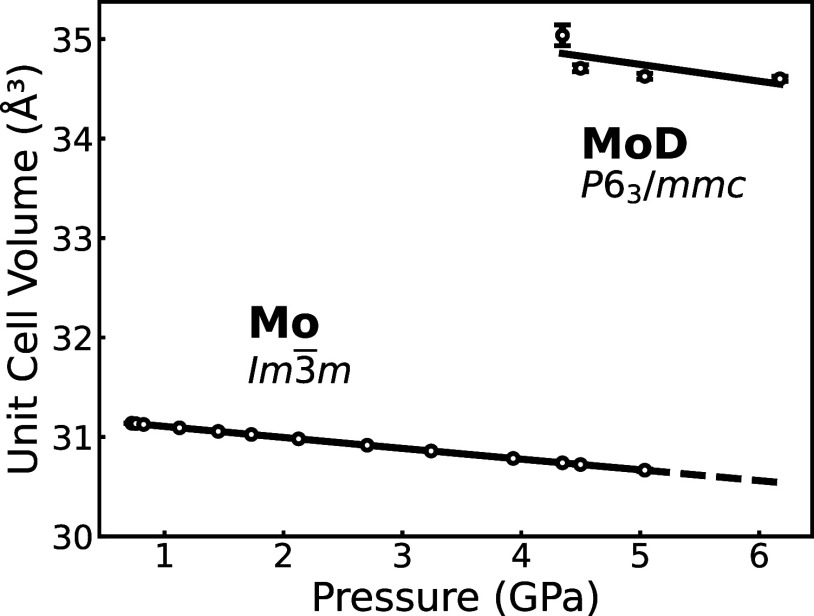
Pressure dependence of the unit-cell volume in the pure Mo metal and MoD_1.15_ component of the diffraction patterns in this study. The trend is illustrated by straight lines as a guide for the eye. Pressure determined from EOS of bcc-Mo is taken from ref 28.

Table 3 presents the refined atomic distances for different pressures in MoD_1.15_. Since the D_T_ atoms are not positioned at the centers of the tetrahedra, as shown in Figure 2, there are two crystallographically inequivalent Mo···D_T_ distances. We refer to these as (1)­Mo···D_T_ and (2)­Mo···D_T_—the first of these is a single interaction aligned with the *z* direction, and the second corresponds to three symmetry-related interactions in the Mo_4_D tetrahedron. The distances between Mo and D atoms reach a minimum of ∼1.49 Å for (1)­Mo···D_T_, while the other three Mo···D_T_ distances are slightly longer, around 1.9 Å. The Mo···D_O_ interactions in the octahedra are crystallographically equivalent, measuring ∼2.06 Å, as D_O_ atoms are located at the high-symmetry sites 2*a*. The relatively short Mo···D_T_ distances (1.49/1.90 Å) indicate that these may represent more substantive interactions than those implied by the longer Mo···D_O_ distance of 2.06 Å. Similar longer-distance distributions are observed in ZrH_1.66_ ^34^ and TiH_2_,^35^ where the metal···H distances are 2.07 Å and 1.93 Å, respectively. In these cases, the H atoms occupy interstitial hydrogen sites, forming multicenter weak interactions with neighboring metal atoms. These interstitial bonds involve a combination of electrostatic interactions and covalent contributions from hydrogen 1*s*-transition metal d-orbital hybridization,^36^ leading to slightly longer bond lengths. As the D_O_ atoms in MoD_*x*_ also sit at interstitial sites, this may be a reasonable explanation for the observed Mo···D_O_ distance of 2.06 Å.

**Table 3:** Atomic Distances of MoD_1.15_ as a Function of Pressure^a^

pressure (GPa)	Mo···D_O_	(1)Mo···D_T_	(2)Mo···D_T_	D_O_···D_O_	D_O_···D_T_	D_T_···D_T_ ^b^
4.35(3)	2.0615(16)	1.497	1.900	2.376(5)	1.714	1.758
4.50(5)	2.0549(5)	1.488	1.895	2.3620(15)	1.709	1.748
5.04(12)	2.0533(5)	1.487	1.894	2.3610(15)	1.708	1.747
6.17^c^	2.0532(5)	1.488	1.893	2.3617(14)	1.707	1.748

a All distances are in Å. Where errors are not shown, this is due to the refinement approach. See Supporting Information for details.

b Note that the D_T_···D_T_ distances refer to intersite distances that are not simultaneously occupied.

c No error is provided as it was inferred from the load-pressure relation. There is no detectable quantity of Mo for reliable pressure determination at this pressure point. Note that the uncertainties on all other pressure points are derived from error propagation in the Rietveld refinements, derived from the Mo equation of state.^28^

In order to place our observed transition metal (TM)–H/D distances in the context of the wider literature, we surveyed the TM··· H/D interatomic distances in transition metal hydrides and deuterides under ambient conditions using data from the Inorganic Crystal Structure Database (ICSD).^37^ The resulting distribution is shown as a red histogram in Figure 6. It can be observed that TM···H/D interatomic distances occur above 1.4 Å, with the majority falling within the range of 1.8–2.2 Å. The second maximum in the distribution, occurring at distances greater than ∼3.2 Å, is likely associated with nonbonded TM and H/D atoms. The three sets of Mo···D distances obtained from our experiments generally align well with this distribution.

**Figure 6:**
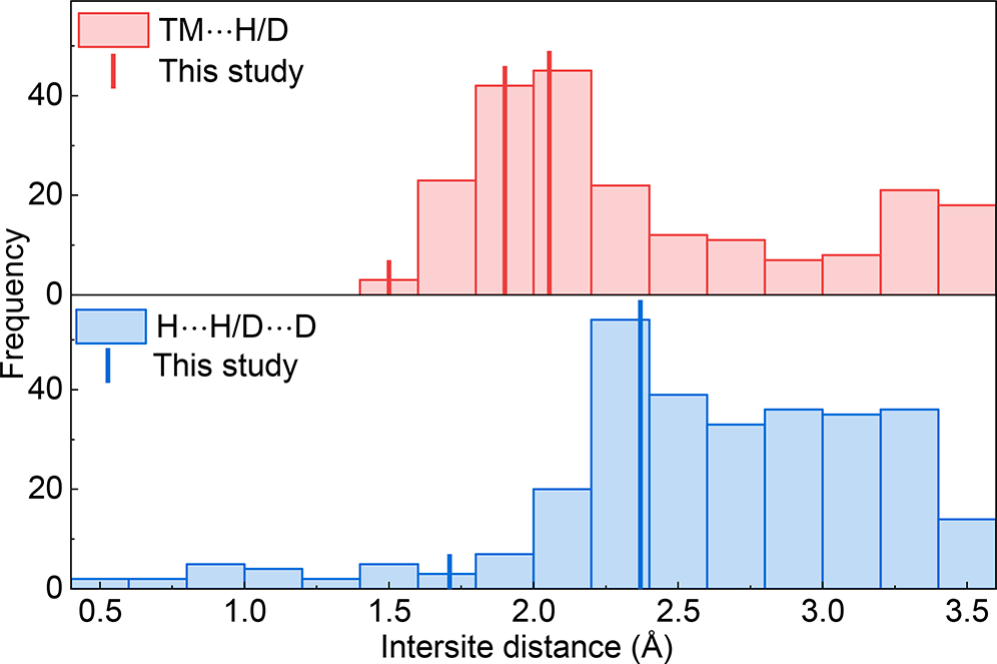
Distributions of interatomic distances in transition metal hydrides and deuterides. The red histogram represents TM···H/D interatomic distances, while the blue histogram represents H···H/D···D intersite distances. Data from the present work are indicated by bold vertical lines. Distribution data obtained from the ICSD.^37^

According to the “empirical 2.1 Å rule”, H···H and D···D distances in transition metal hydrides/deuterides tend to remain above 2.1 Å due to electronic structure constraints,^38^ indicating that H/D atoms are unlikely to exist as molecular H_2_/D_2_. The distribution of H···H/D···D intersite distances in transition metal hydrides/deuterides obtained from the ICSD (represented by the blue histogram in Figure 6) qualitatively reproduces this behavior, showing a clear step change above ca. 2.1 Å. We note that these distributions do not represent a direct measure of H···H/D···D distances—rather the crystallographic sites they (may) occupy—this is clearly the case for distances <2.0 Å. Experimentally, only a limited number of transition metal hydrides/deuterides have been reported to exhibit short H···H/D···D distances that violate the empirical 2.1 Å rule.^24,39–41^ Our D_O_···D_O_ distances follow the 2.1 Å rule, while the D_O_···D_T_ distances of ∼1.7 Å are relatively shorter. The strong repulsion caused by short D···D distances explains the preferential occupation of the octahedral site: any higher occupancy of the tetrahedral sites would result in more unfavorable short D···D distances.

## Conclusion

In conclusion, the formation of MoD_1.15_ was observed to begin through direct interaction with D_2_ gas at pressures between 3.9 and 4.4 GPa. By performing neutron diffraction on MoD_1.15_, we determined the atomic positions and occupancies and confirmed the *P*6_3_/*mmc* symmetry. Our results show that D atoms nearly fully occupy the octahedral sites and partially occupy the tetrahedral sites. The refined atomic positions and the surveyed interatomic distances in transition metal hydrides/deuterides provide a clearer picture of the interatomic interactions, suggesting the presence of relatively strong interaction between Mo and D_T_ and multicenter weak interactions between Mo and D_O_. The effect of D···D repulsion explains the partial occupancy of tetrahedral sites and, thus, the nonstoichiometric D:Mo value in MoD_1.15_. These findings provide insights into the synthesis and structural properties of hydrogen-rich transition metal hydrides and demonstrate the unique information that neutron diffraction can offer for related studies.

## Supplementary Material



## Footnotes

a The pressures are determined by fitting the equation of state of Mo,^28^ while the 6.2 GPa value is inferred from the load-pressure relation.

## Notes

Model crystallographic parameters; simulated neutron diffraction patterns and relative intensity analysis; refinement strategy; multidimensional heat map of site occupancies and tetrahedral site *z*-coordinate; and Rietveld refinements for other models and pressure points (PDF)

## ∥

Neutron Scattering Division, Oak Ridge National Laboratory, Oak Ridge, TN 37831, USA

## References

[1] Klopčič N., Grimmer I., Winkler F., Sartory M., Trattner A. (2023). A Review on
Metal Hydride Materials for Hydrogen Storage. J. Energy Storage..

[2] Tarasov B. P., Fursikov P. V., Volodin A. A., Bocharnikov M. S., Shimkus Y. Y., Kashin A. M., Yartys V. A., Chidziva S., Pasupathi S., Lototskyy M. V. (2021). Metal Hydride
Hydrogen Storage and
Compression Systems for Energy Storage Technologies. Int. J. Hydrogen Energy.

[3] Trattner A., Höglinger M., Macherhammer M.-G., Sartory M. (2021). Renewable Hydrogen:
Modular Concepts from Production Over Storage to the Consumer. Chem. Eng. Technol..

[4] Antonov V. E. (2002). Phase Transformations,
Crystal and Magnetic Structures of High-pressure Hydrides of D-metals. J. Alloys Compd..

[5] Lang J., Huot J. (2011). A New Approach to the
Processing of Metal Hydrides. J. Alloys Compd..

[6] Sugimoto H., Fukai Y. (1992). Solubility of hydrogen
in metals under high hydrogen pressures: Thermodynamical
calculations. Acta Metall. Mater..

[7] Atou T., Badding J. (1995). In Situ Diffraction
Study of the Formation of Rhenium
Hydride at High Pressure. J. Solid State Chem..

[8] Scheler T., Degtyareva O., Gregoryanz E. (2011). On the Effects
of High Temperature
and High Pressure on the Hydrogen Solubility In Rhenium. J. Chem. Phys..

[9] Scheler T., Degtyareva O., Marqués M., Guillaume C. L., Proctor J. E., Evans S., Gregoryanz E. (2011). Synthesis
and Properties of Platinum Hydride. Phys. Rev.
B.

[10] Kuzovnikov M. A., Tkacz M. (2016). Synthesis of Ruthenium Hydride. Phys. Rev.
B.

[11] Baranowski, B. ; Wisniewski, R. Formation of Nickel Hydride from Nickel and Gaseous Hydrogen. Bull. Acad. Pol. Sci., Ser. Sci. Chim. 1966, 14.

[12] Besedin S.
P., Jephcoat A. P. (1998). High-pressure
X-ray Studies of the Ni-H, Re-H and Al-H
Systems. Rev. High Pressure Sci. Technol..

[13] Kuzovnikov M. A., Meng H., Tkacz M. (2017). Nonstoichiometric Molybdenum
Hydride. J. Alloys Compd..

[14] Belash I. T., Antonov V. E., Ponyatovskij E. G. (1977). Preparation
of Molybdenum Hydride
at High Hydrogen Pressure. Dokl. Akad. Nauk
SSSR.

[15] Antonov V.
E., Belash I. T., Zharikov O. V., Latynin A. I., Palnichenko A. V. (1988). Superconductivity
of Molybdenum Hydride and Deuteride. Fiz. Tverd.
Tela.

[16] Scheler T., Peng F., Guillaume C. L., Howie R. T., Ma Y., Gregoryanz E. (2013). Nanocrystalline
Tungsten Hydrides at High Pressures. Phys. Rev.
B.

[17] Ying J. J., Liu H., Greenberg E., Prakapenka V. B., Struzhkin V. V. (2018). Synthesis
of New Nickel Hydrides at High Pressure. Phys.
Rev. Mater..

[18] Binns J., Donnelly M.-E., Wang M., Hermann A., Gregoryanz E., Dalladay-Simpson P., Howie R. T. (2018). Synthesis of Ni_2_H_3_ at High Temperatures
and Pressures. Phys. Rev. B.

[19] Binns J., He Y., Donnelly M.-E., Peña-Alvarez M., Wang M., Kim D. Y., Gregoryanz E., Dalladay-Simpson P., Howie R. T. (2020). Complex Hydrogen
Substructure in Semimetallic RuH_4_. J. Phys. Chem. Lett..

[20] Marizy A., Geneste G., Loubeyre P., Guigue B., Garbarino G. (2018). Synthesis
of Bulk Chromium Hydrides Under Pressure of up to 120 GPa. Phys. Rev. B.

[21] Antonov V. E., Latynin A. I., Tkacz M. (2004). T–P
Phase Diagrams and Isotope
Effects in the Mo–H/D Systems. J. Phys.:
Condens. Matter.

[22] Fukai Y., Mizutani M. (2003). The Phase Diagram of
Mo-H Alloys Under High Hydrogen
Pressures. Mater. Trans..

[23] Abramov S. N., Antonov V. E., Bulychev B. M., Fedotov V. K., Kulakov V. I., Matveev D. V., Sholin I. A., Tkacz M. (2016). T–P
Phase Diagram
of the Mo–H System Revisited. J. Alloys
Compd..

[24] Kuzovnikov M. A., Antonov V. E., Hansen T., Ivanov A. S., Kolesnikov A. I., Kulakov V. I., Muzalevsky V. D., Savvin S., Tkacz M. (2022). Isotopic Dependence
of the Frequency of Optical Vibrations in Molybdenum Monohydride. J. Alloys Compd..

[25] Feng X., Zhang J., Liu H., Iitaka T., Yin K., Wang H. (2016). High Pressure Polyhydrides
of Molybdenum: A First-principles Study. Solid
State Commun..

[26] Irodova A. V., Glazkov V. P., Somenkov V. A., Shil’shtejn S. S., Antonov V. E., Ponyatovskij E. G. (1988). Neutron-diffraction
Study of Molybdenum,
Rhodium, and Nickel Hydride Structure. Kristallografiya.

[27] Klotz S., Philippe J., Bull C. L., Loveday J. S., Nelmes R. J. (2013). A 3 kbar
Hydrogen-compatible Gas Loader for Paris–Edinburgh Presses. High Pressure Res..

[28] Huang X., Li F., Zhou Q., Meng Y., Litasov K. D., Wang X., Liu B., Cui T. (2016). Thermal Equation of State of Molybdenum Determined
from in situ Synchrotron X-ray Diffraction With Laser-heated Diamond
Anvil Cells. Sci. Rep..

[29] Bull C. L., Funnell N. P., Tucker M. G., Hull S., Francis D. J., Marshall W. G. (2016). Pearl: The High
Pressure Neutron Powder Diffractometer
at ISIS. High Pressure Res..

[30] Arnold O., Bilheux J. C., Borreguero J. M., Buts A., Campbell S. I., Chapon L., Doucet M., Draper N., Ferraz-Leal R., Gigg M. A., Lynch V. E., Markvardsen A., Mikkelson D. J., Mikkelson R. L., Miller R., Palmen K., Parker P., Passos G., Perring T. G., Peterson P. F., Ren S., Reuter M. A., Savici A. T., Taylor J. W., Taylor R. J., Tolchenov R., Zhou W., Zikovsky J. (2014). Mantid—Data
Analysis and Visualization Package for Neutron Scattering and μsr
Experiments. Nucl. Instrum. Methods Phys. Res.,
Sect. A.

[31] Coelho, A. A. Topas V6.0: Brisbane, Australia, 2015.

[32] Kuzovnikov M. A., Antonov V. E., Ivanov A. S., Hansen T., Savvin S., Kulakov V. I., Tkacz M., Kolesnikov A. I., Gurev V. M. (2020). Neutron Scattering Study of Tantalum
Dihydride. Phys. Rev. B.

[33] Fukai, Y. The Metal-Hydrogen System: Basic Bulk Properties; Springer Berlin Heidelberg: Berlin, Heidelberg, 2005; pp 91–147.

[34] Maimaitiyili T., Steuwer A., Blomqvist J., Bjerkén C., Blackmur M. S., Zanellato O., Andrieux J., Ribeiro F. (2016). Observation
of the δ to ε Zr-hydride Transition by In-situ Synchrotron
X-ray Diffraction. Cryst. Res. Technol..

[35] Kalita P. E., Sinogeikin S. V., Lipinska-Kalita K., Hartmann T., Ke X., Chen C., Cornelius A. (2010). Equation of
State of TiH_2_ up to 90 GPa: A Synchrotron X-ray Diffraction
Study and ab initio
Calculations. J. Appl. Phys..

[36] Nørskov J. K. (1982). Covalent
Effects in the Effective-medium theory of Chemical Binding: Hydrogen
Heats of Solution in the 3d Metals. Phys. Rev.
B.

[37] FIZ Karlsruhe – Leibniz Institute for Information Infrastructure Inorganic Crystal Structure Database (icsd). 2024; https://icsd.fiz-karlsruhe.de.

[38] Switendick A. C. (1979). Band Structure
Calculations for Metal Hydrogen Systems. Z.
Phys. Chem..

[39] Klein R. A., Balderas-Xicohténcatl R., Maehlen J. P., Udovic T. J., Brown C. M., Delaplane R., Cheng Y., Denys R. V., Ramirez-Cuesta A. J., Yartys V. A. (2022). Neutron Vibrational Spectroscopic
Evidence for Short H··· H Contacts in the RNiInH_1.4;1.6_ (R = Ce, La) Metal Hydride. J.
Alloys Compd..

[40] Kuzovnikov M. A., Antonov V. E., Kulakov V. I., Muzalevsky V. D., Orlov N. S., Palnichenko A. V., Shulga Y. M. (2023). Synthesis of Superconducting
hcp-ZrH_3_ Under High Hydrogen Pressure. Phys. Rev. Mater..

[41] Borgschulte A., Terreni J., Billeter E., Daemen L., Cheng Y., Pandey A., Łodziana Z., Hemley R. J., Ramirez-Cuesta A. J. (2020). Inelastic
Neutron Scattering Evidence for Anomalous H–H Distances in
Metal Hydrides. Proc. Natl. Acad. Sci. U.S.A..

[42] Bull, C. L. ; Ridley, C. J. ; Funnell, N. P. ; Pulham, C. R. ; Klotz, S. Formation and Structural Properties of Non-stoichiometric Molybdenum Hydride, 2019; https://data.isis.stfc.ac.uk/doi/INVESTIGATION/103216662/.

