# Supplementary material for: Formation of Molybdenum Deuteride at High Pressure: A Neutron Diffraction Study
Source: Inorg Chem. 2025 Dec 18;65(1):631–7. doi: 10.1021/acs.inorgchem.5c04811 (PMC12801321; doi:10.1021/acs.inorgchem.5c04811)
Supplement: Supplementary file 1 [file ic5c04811_si_001.pdf]

Supporting Information: Formation of Molybdenum Deuteride at High Pressure: A Neutron Diffraction Study

Zhongsheng Wei,[†] Nicholas P. Funnell,[†] Christopher J. Ridley,[†,§] Stefan Klotz,[‡] Colin R. Pulham,[¶] and Craig L. Bull[*,†,¶]

†ISIS Neutron and Muon Facility, Rutherford Appleton Laboratory, Didcot OX11 0QX, UK.

‡Institut de Minéralogie, de Physique des Matériaux et de Cosmochimie, Sorbonne Université, UMR CNRS 7590, Paris, France.

¶School of Chemistry, University of Edinburgh, David Brewster Road, Edinburgh EH9 3FJ, Scotland, UK

§Now at: Neutron Scattering Division, Oak Ridge National Laboratory, Oak Ridge, TN 37831, USA.

E-mail: craig.bull@stfc.ac.uk

Model Parameterisation

Table S1: Lattice and atomic parameters of $MoD_{1+\delta} (0 \leq \delta \leq 1)$ in simulated neutron diffraction pattern*.

Model	Space group	a (Å)	c (Å)	Atom	x	y	z	Occupancy	beq (Å^2)
model 1	$P6_3mc$	2.91	4.72	Mo	1/3	2/3	1/4	1	1
				D_O	0	0	0	1	1.5
				D_T	1/3	2/3	0.63	δ	1.5
model 2	$P6_3/mmc$	2.91	4.72	Mo	1/3	2/3	1/4	1	1
				D_O	0	0	0	1	1.5
				D_T	1/3	2/3	0.63	$\delta/2$	1.5
model 3	$P6_3mc$	2.91	4.72	Mo	1/3	2/3	1/4	1	1
				D_O	0	0	0.383 †	1	1.5
				D_T	1/3	2/3	0.63	0	1.5

* Simulations were conducted to evaluate the sensitivity of reflections to the choice of space group (model 1 and model 2) and to the displacement of the D_O atom (model 3).
† A shift of -0.117 is applied, as suggested by a previous simulation study on molybdenum hydrides.[S1]

Table S2: Lattice and atomic parameters of $MoD_{\zeta} (0 \leq \zeta \leq 1)$ in simulated neutron diffraction patterns*.

Model	Space group	a (Å)	c (Å)	Atom	x	y	z	Occupancy	beq (Å^2)
model 4	$P6_3mc$	2.91	4.72	Mo	1/3	2/3	1/4	1	1
				D_O	0	0	0	ζ or 0	1.5
				D_T	1/3	2/3	0.63	0 or ζ	1.5
model 5	$P6_3/mmc$	2.91	4.72	Mo	1/3	2/3	1/4	1	1
				D_O	0	0	0	ζ or 0	1.5
				D_T	1/3	2/3	0.63	0 or $\zeta/2$	1.5

* Simulations were conducted to evaluate the sensitivity of reflections to the occupancies of D atoms in the $P6_3mc$ structure (model 4) and the $P6_3/mmc$ structure (model 5). Results presented in Fig. S2.

Results of Simulated Neutron Diffraction Patterns

Simulated diffraction patterns were calculated using Topas Academic V6.[S2] Only a single phase (MoD) was considered, and the structural parameters used for the simulations are

provided in Tables S1 and S2. Peak profiles were defined using a time-of-flight pseudo-voigt description with parameters fixed to typical values seen on the PEARL diffractometer at ISIS.

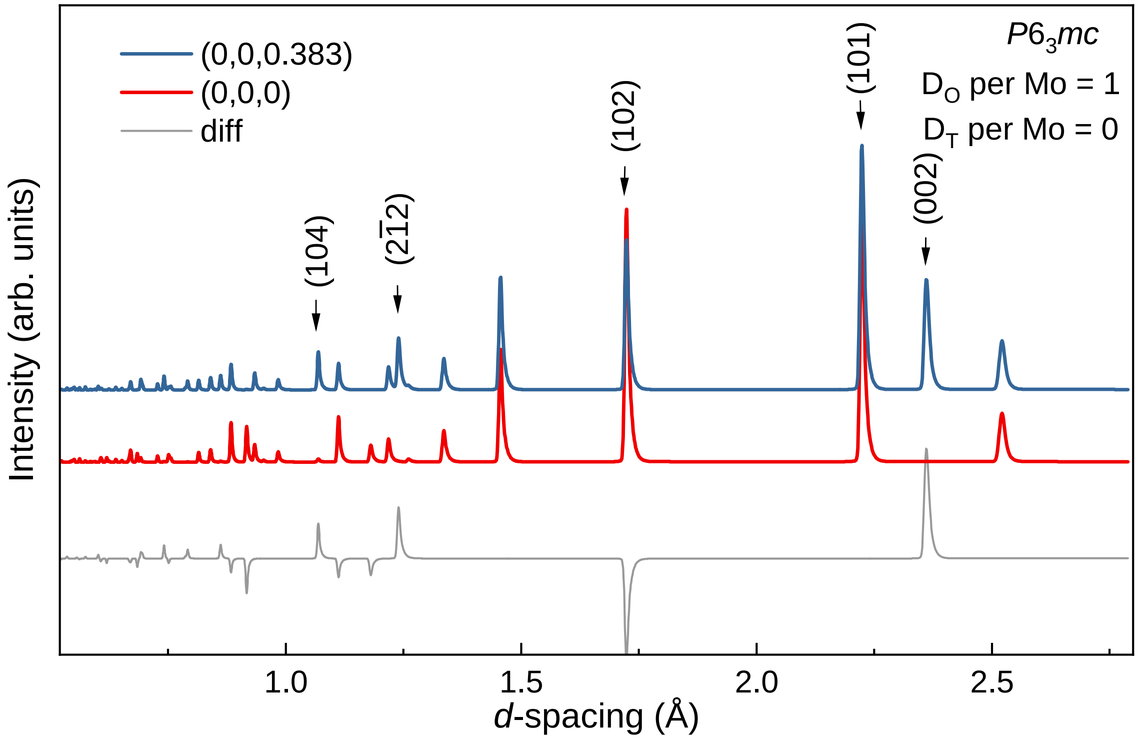

Figure S1: Effect of D_O atom displacement on neutron diffraction patterns. The blue pattern represents the simulation result of MoD with a -0.117 shift in the fractional z-coordinate of the D_O atom. The red pattern represents the simulation result of MoD_x with the D_O atom remaining at the centre of the octahedron. The grey line shows the difference between the two patterns. The (*hkl*) reflections with noticeable intensity changes are marked with arrows.

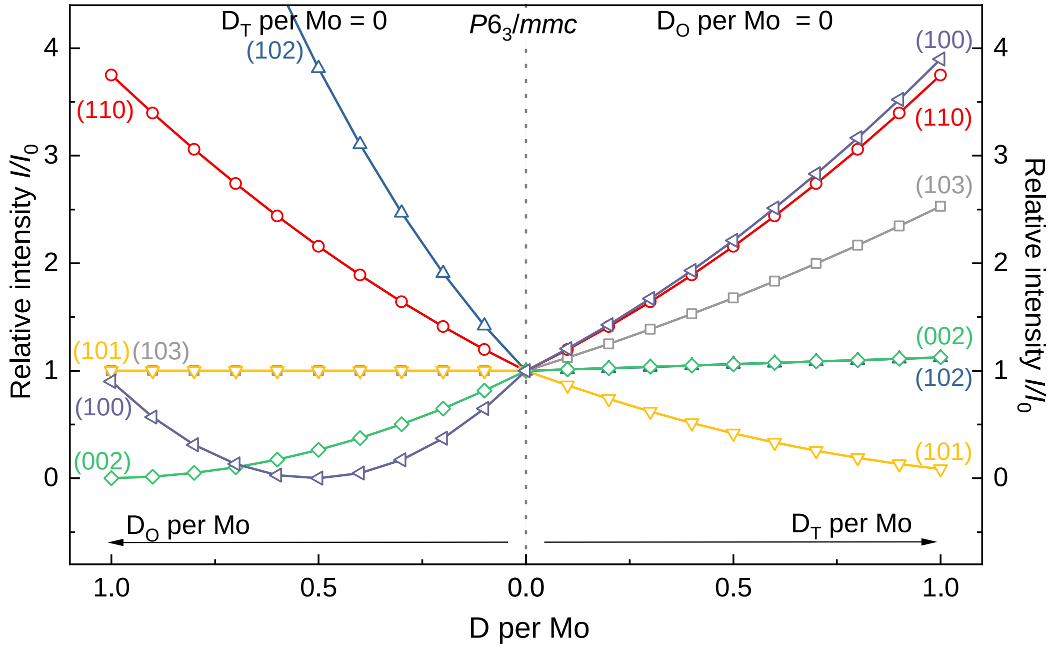

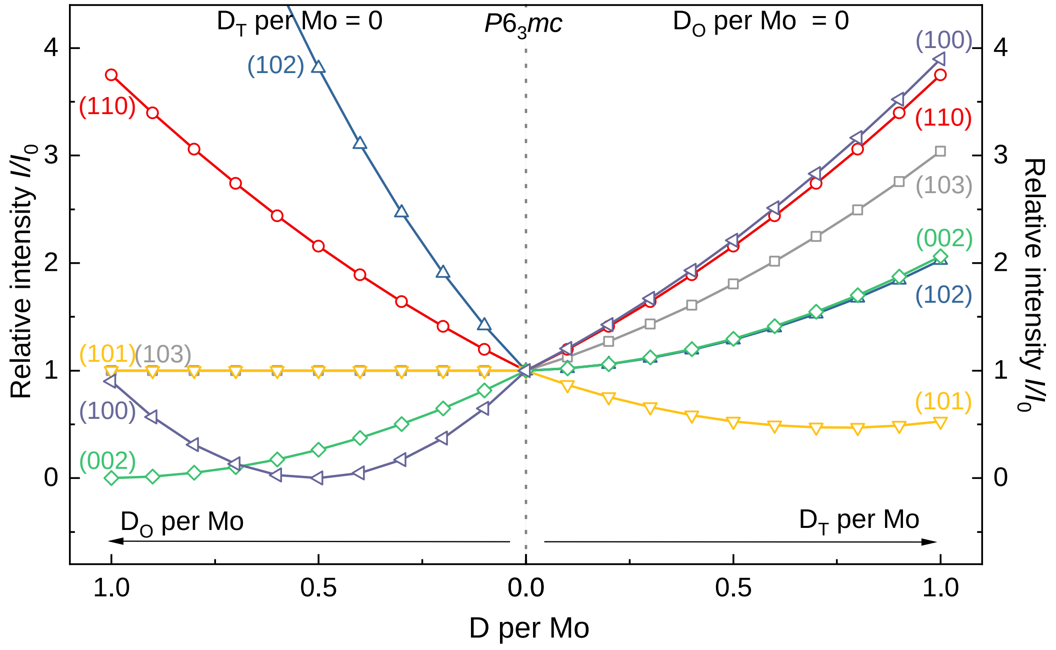

Figure S2: Effect of D_O and D_T content variations on the relative intensities (I/I_0, where I_0 is the intensity at D/Mo = 0). Simulations were performed using the space groups $P6_3/mmc$ (top) and $P6_3mc$ (bottom). In the left panel of each plot, D_O per Mo varies from 0 to 1, while D_T per Mo is fixed at 0. In the right panel, D_T per Mo varies from 0 to 1, with D_O per Mo set to 0. Different colours represent different reflections, with the corresponding (*hkl*) indices labeled at the end of each curve.

Rietveld Refinements of $P6_3mc$ and $Pnma$ Models at 6.2 GPa

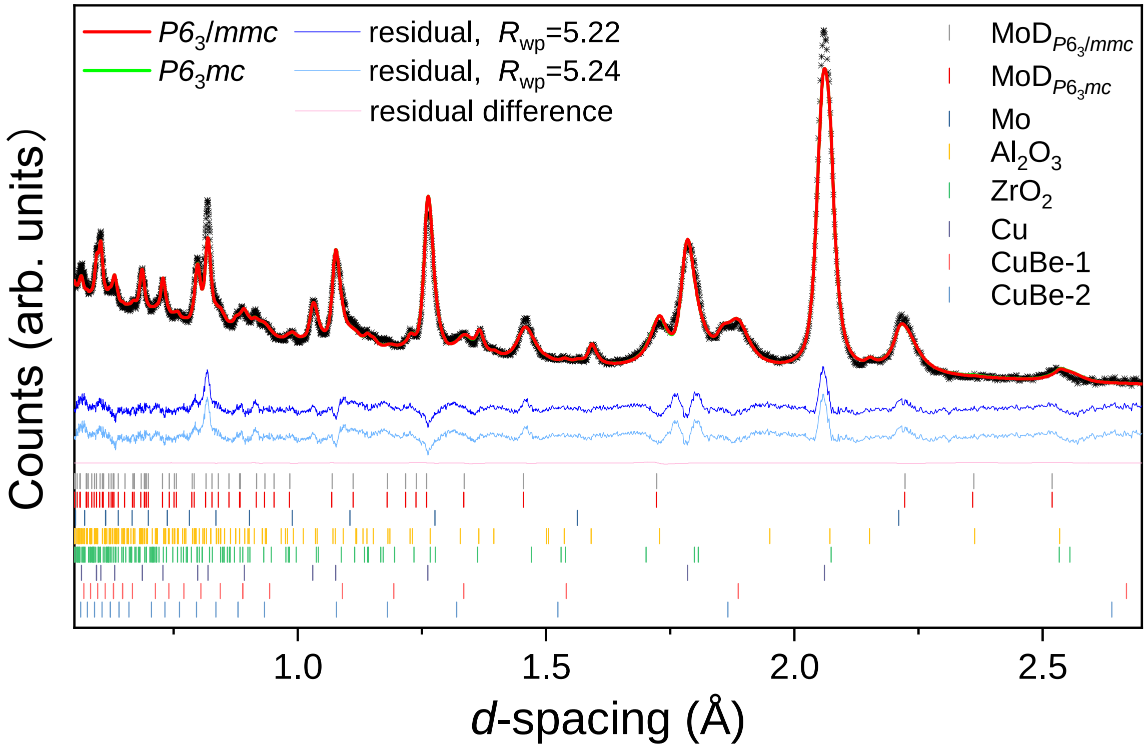

Figure S3: The comparison of neutron powder diffraction patterns of MoD_x using different space groups, $P6_3/mmc$ and $P6_3mc$, at 6.2 GPa. The Rietveld fits are represented as red ($P6_3/mmc$) and green ($P6_3mc$) lines, while the green line is overlaid by the red line due to the similar fit quality. Experimental data are represented by black marks. The residuals are shown as two blue lines with offset for clearer view. The difference between residuals is presented as pink line for comparison. Vertical tickmarks indicate the positions of reflections from MoD_x, Mo, anvils (Al_2O_3 and ZrO_2) and gasket (Cu and CuBe), respectively. Note that the two CuBe components correspond to the same impurity phase at two different scattering locations.

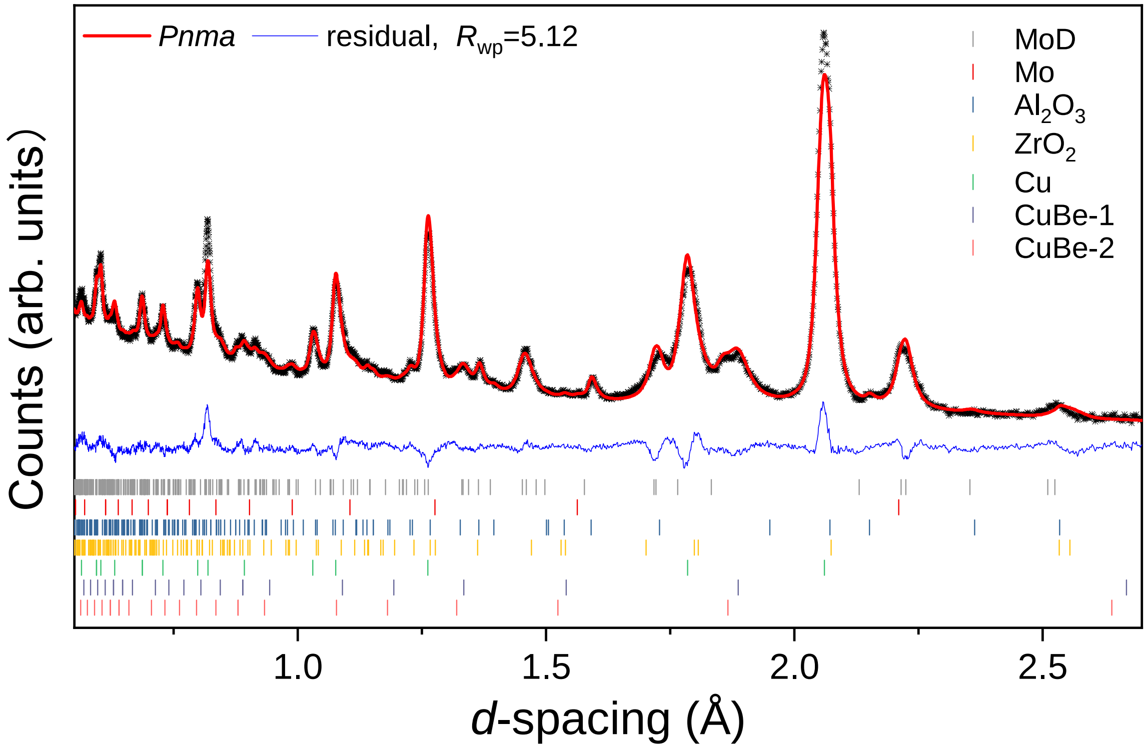

Figure S4: Neutron powder diffraction patterns of MoD_x using the space group *Pnma* at 6.2 GPa .[S3] The Rietveld fit is represented as a red line, while the experimental data are represented by black marks. The residual is shown as a blue line. Vertical tickmarks indicate the positions of reflections from MoD_x, Mo, anvils (Al_2O_3 and ZrO_2) and gasket (Cu and CuBe), respectively. The quality of fit does not warrant lowering the symmetry below $P6_3/mmc$. Note that the two CuBe components correspond to the same impurity phase at two different scattering locations.

Rietveld Refinements Strategy and Results

Rietveld refinements were performed using Topas Academic V6.[S2] For pressures $\leq$ 3.9 GPa, Rietveld refinements were performed on five phases: Mo, Al_2O_3, ZrO_2, Cu and CuBe. The CuBe phase was included as two components, corresponding to the same impurity scattering from two different locations of the gasket. These impurity reflections were also observed in the empty clamp (see Fig. S8). Each phase, including the two CuBe components, was fitted using an independent peak shape function. Preferred orientation was refined for the Al_2O_3 and Cu phases at one of the most reliable data points (longest collection and best statistics, 1.4 GPa) and then held fixed throughout the experiment for consistency. An isotropic displacement parameter for the Mo atom was determined from refinement of Mo powder in a V can (see Fig. S7), and this fixed value was used for the refinements at pressure.

For pressures above 3.9 GPa, MoD phase was added to the refinements. We first performed conventional Rietveld refinement on all six phases. Due to the limited amount of MoD synthesised for the first few pressure points and the complexity of the diffraction pattern, we could not get stable refinements of the D atom site occupancies and the tetrahedral site z-coordinates. To identify the most favourable values of these, we systematically varied each parameter within their physically reasonable limits using the 6.2 GPa dataset, and plotted R_{wp} as a function of them, resulting in a multi-dimensional heat map. The heat map and corresponding parameter ranges are shown in Fig. S5, with the region of favourable parameter space highlighted by a blue rectangular box. A finer mapping was then performed within this region (see Fig. S6), providing the optimal D atom site occupancies and z-coordinates of the tetrahedral site. Based on the discussion in the main manuscript, we consider the D content to remain unchanged across the four pressure points, and therefore these D atom parameters were used for all refinements.

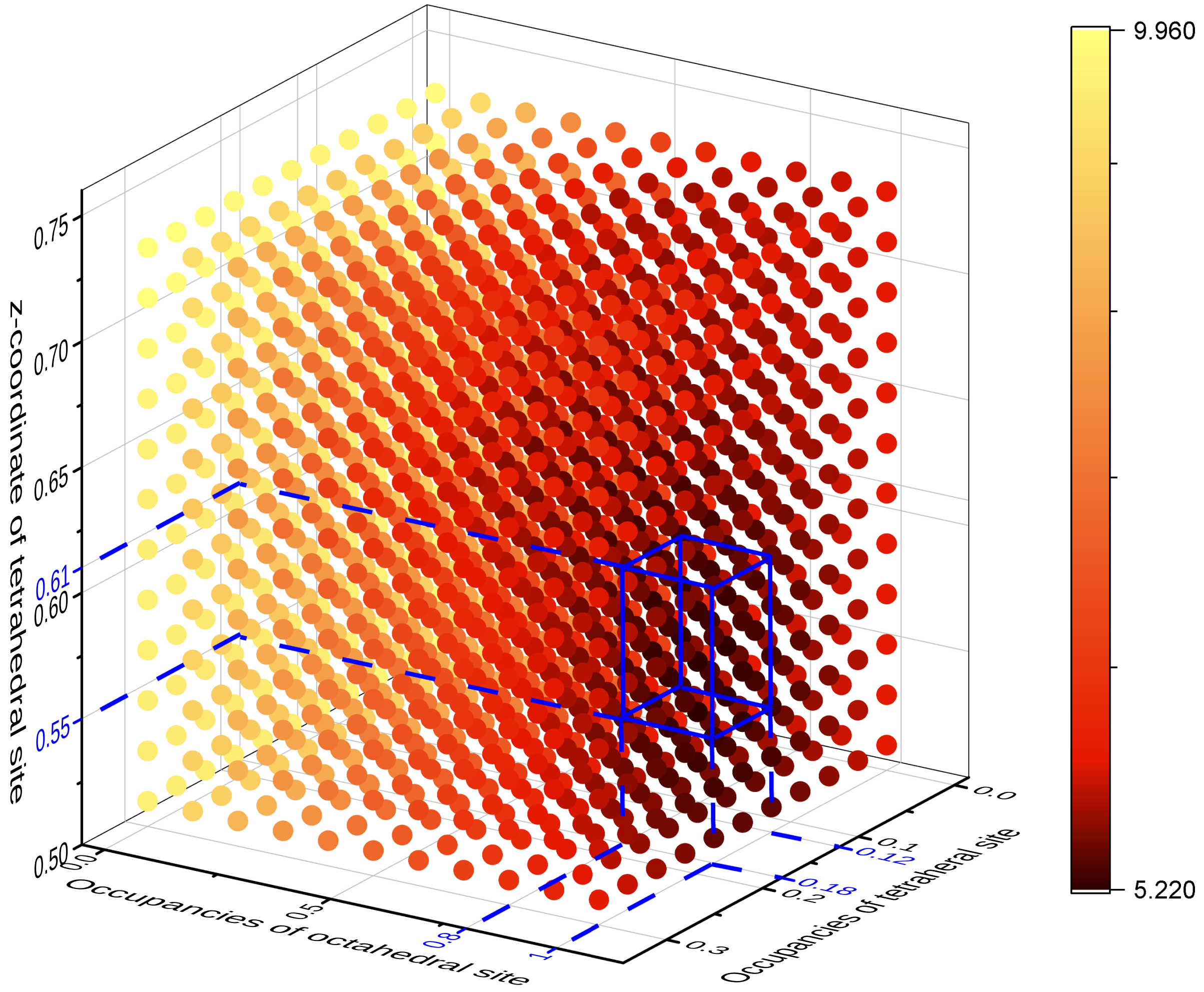

Figure S5: Multi-dimensional heat map of D atom occupancies and tetrahedral site z-coordinate for the MoD phase at 6.2 GPa. The parameter ranges and step sizes are: 0.0–1.0 (step 0.1) for the octahedral site occupancy; 0.00–0.30 (step 0.03) for the tetrahedral site occupancy; and 0.51–0.73 (step 0.02) for the tetrahedral site z-coordinate. The blue rectangular box indicates the region of favourable parameter space. The colour scale represents R_{wp} (%).

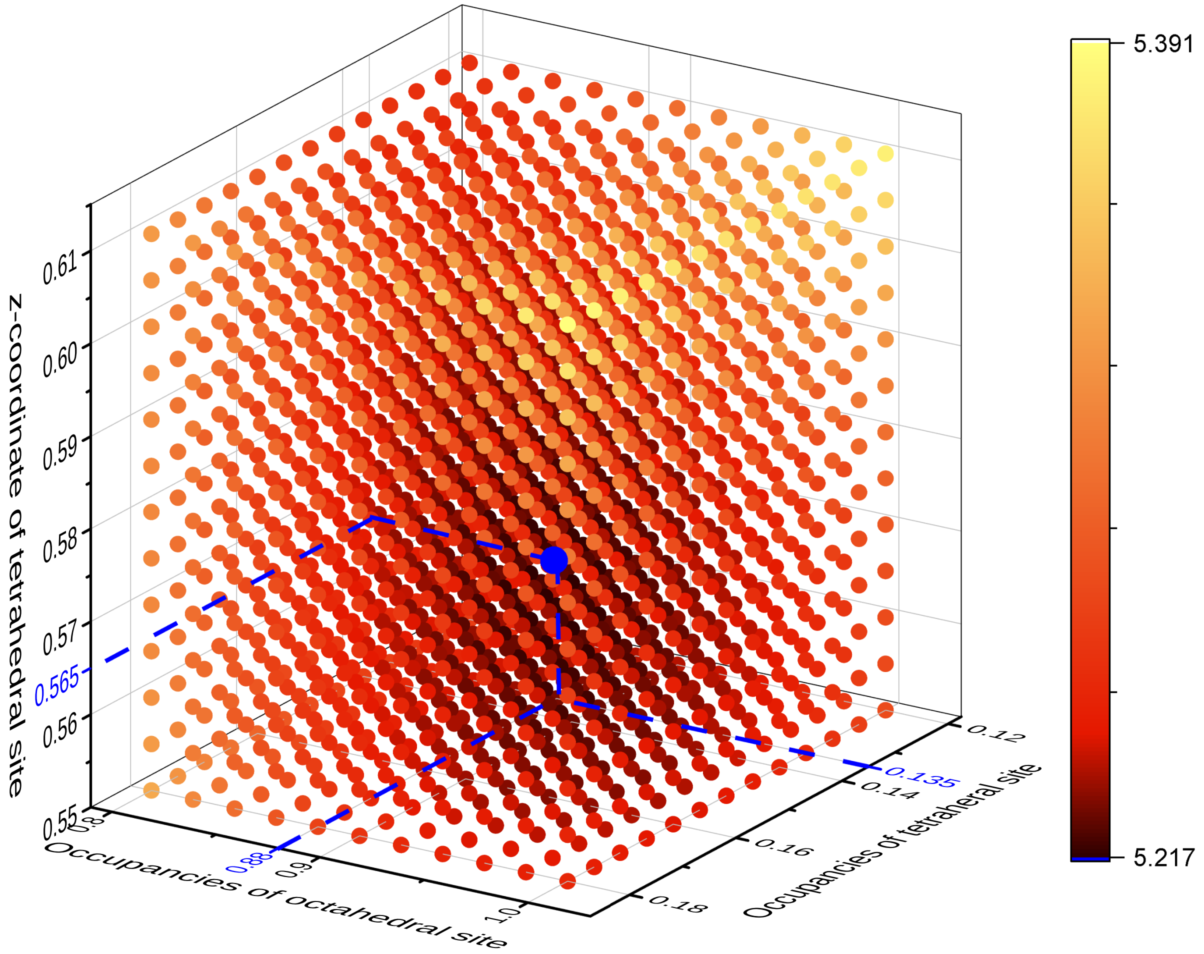

Figure S6: Multi-dimensional heat map of D atom occupancies and tetrahedral site z-coordinate in the region of favourable parameter space for the MoD phase at 6.2 GPa. The parameter ranges and step sizes are: 0.80–1.00 (step 0.02) for the octahedral site occupancy; 0.120–0.180 (step 0.005) for the tetrahedral site occupancy; and 0.550–0.610 (step 0.005) for the tetrahedral site z-coordinate. The point with the lowest R_{wp} is highlighted in blue. The colour scale represents R_{wp} (%).

Rietveld Refinements for Empty Clamp, Mo powder, and All Pressure Points

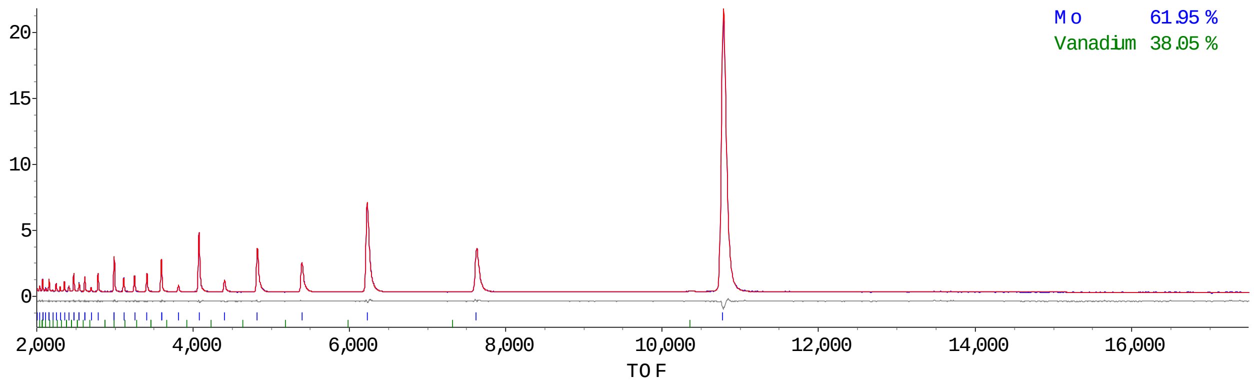

Figure S7: Rietveld refinement of neutron powder diffraction experiment for Mo powder in a V can at ambient conditions. The y-axis is the relative intensities. On the PEARL instrument, d-spacing $\approx$ TOF/4842.

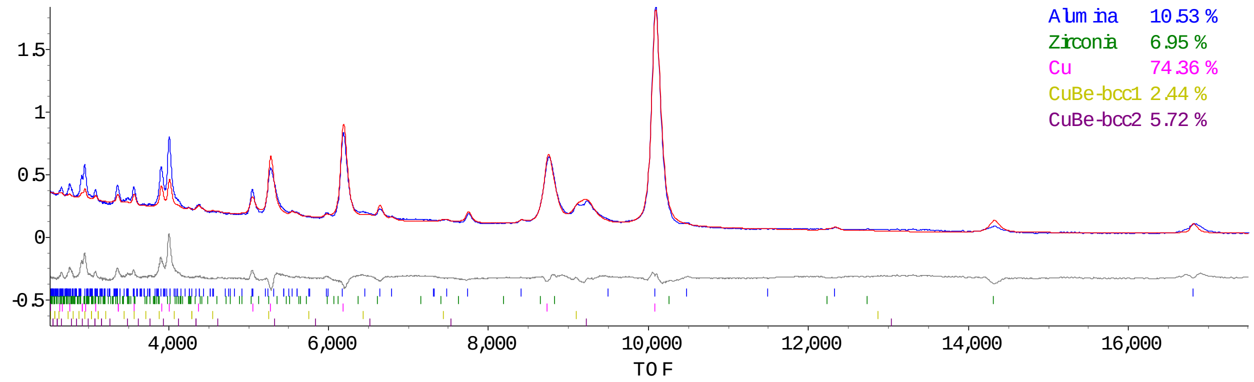

Figure S8: Rietveld refinement of neutron powder diffraction experiments for empty clamp. The y-axis is the relative intensities. On the PEARL instrument, d-spacing $\approx$ TOF/4842.

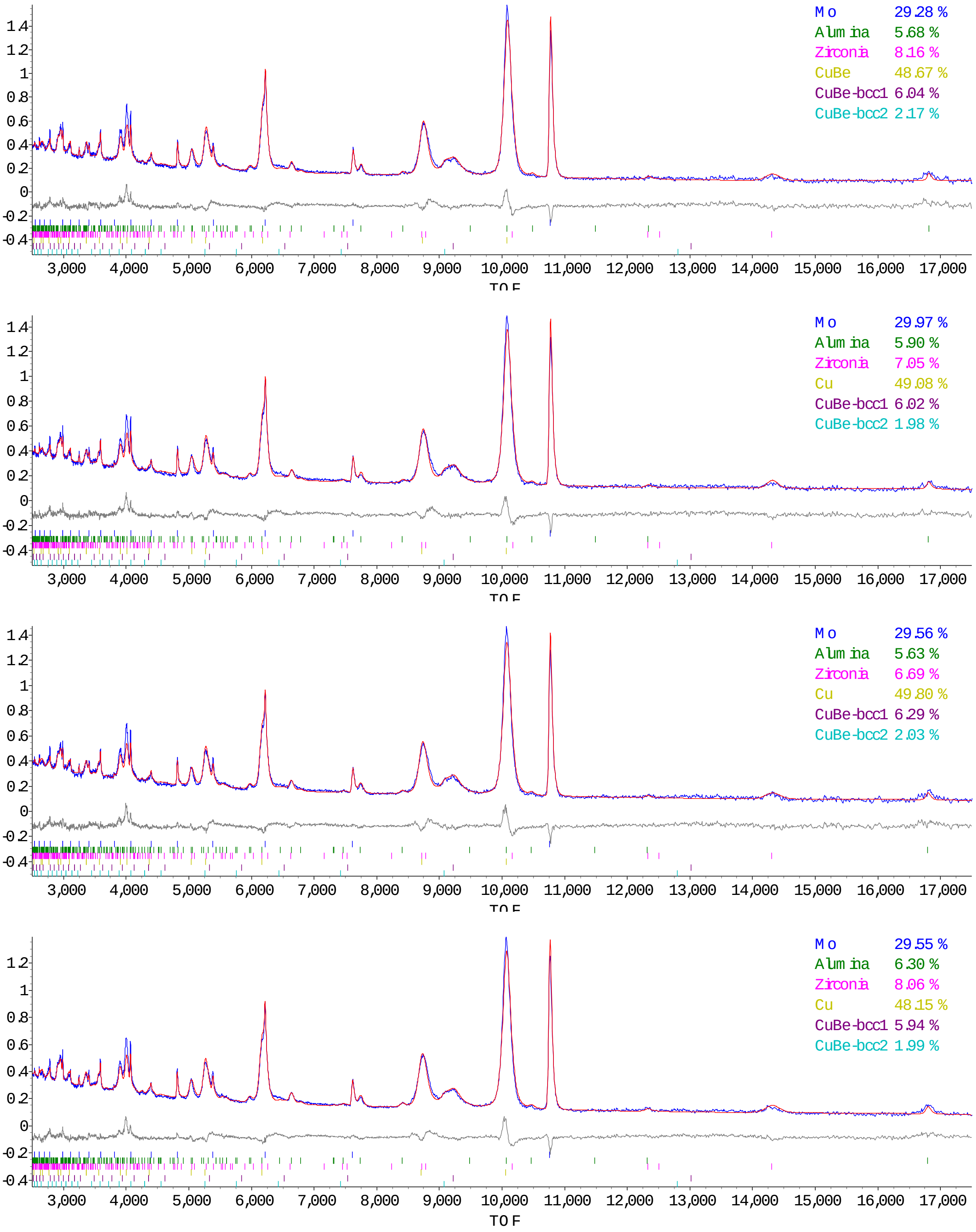

Figure S9: Rietveld refinement results of neutron powder diffraction experiments for (from top to bottom) 0.7, 0.8, 1.1 and 1.5 GPa. The y-axes are the relative intensities. On the PEARL instrument, d-spacing $\approx$ TOF/4842.

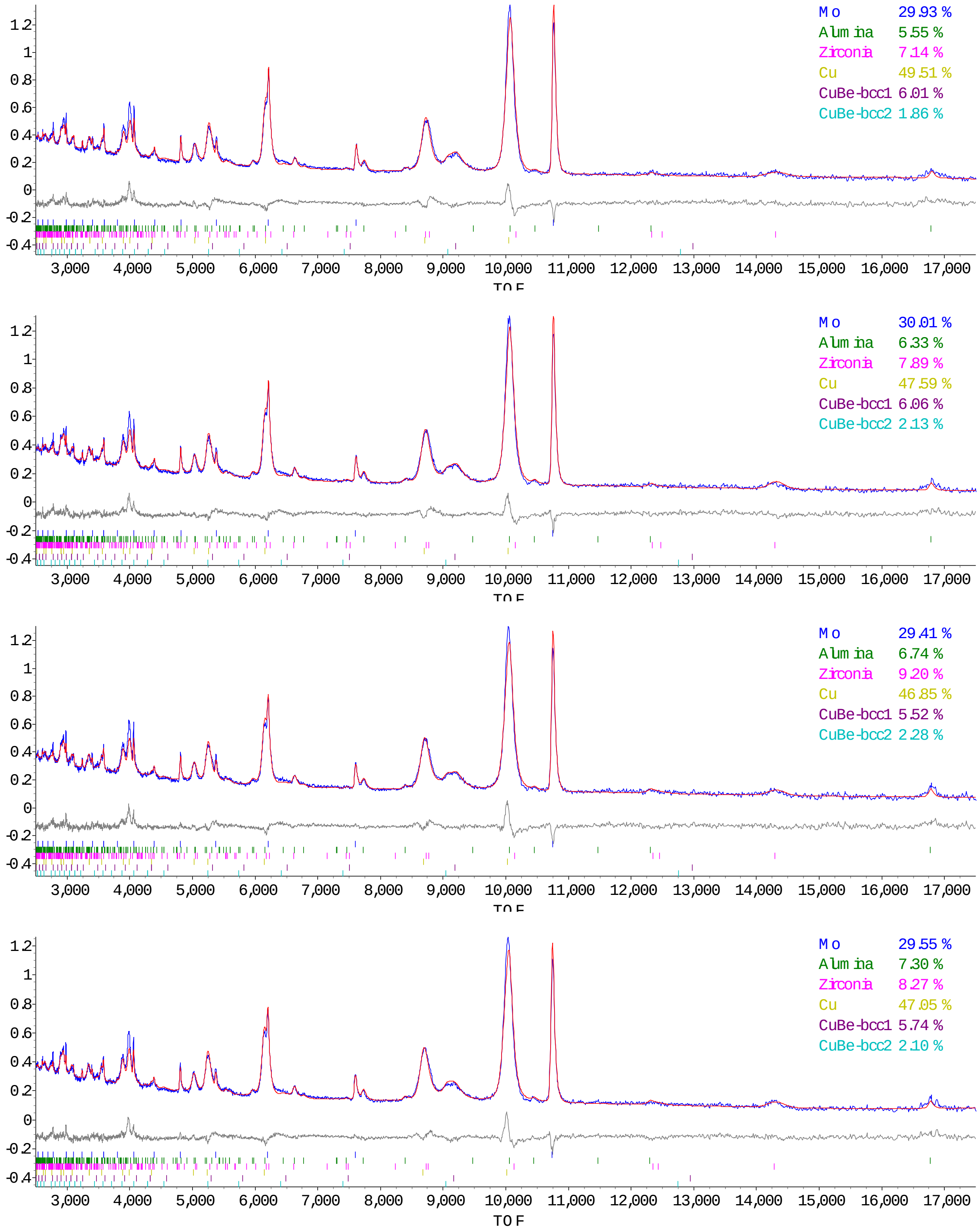

Figure S10: Rietveld refinement results of neutron powder diffraction experiments for (from top to bottom) 1.7, 2.1, 2.7 and 3.2 GPa. The y-axes are the relative intensities. On the PEARL instrument, d-spacing $\approx$ TOF/4842.

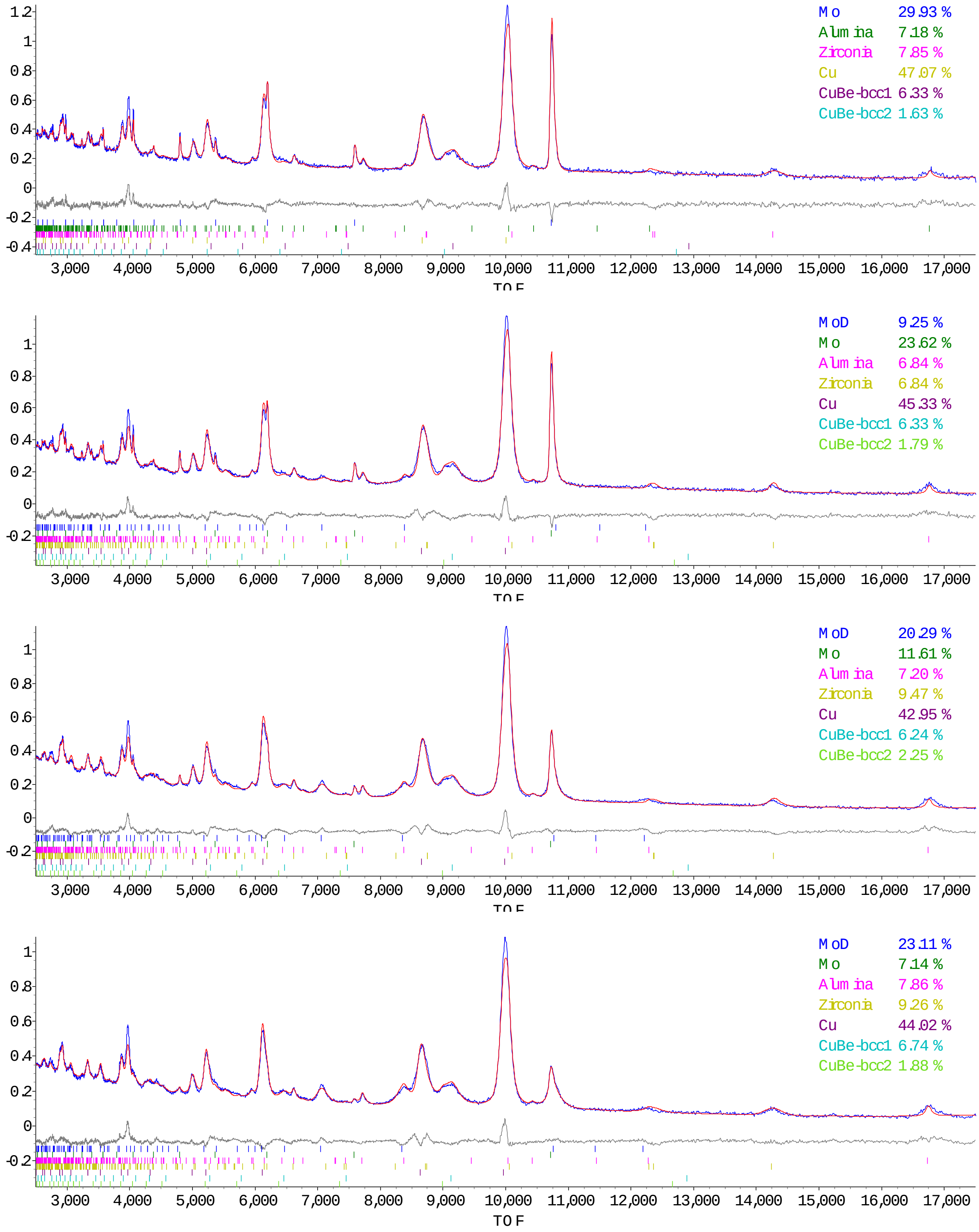

Figure S11: Rietveld refinement results of neutron powder diffraction experiments for (from top to bottom) 3.9, 4.4, 4.5 and 5.0 GPa. The y-axes are the relative intensities. On the PEARL instrument, d-spacing $\approx$ TOF/4842.

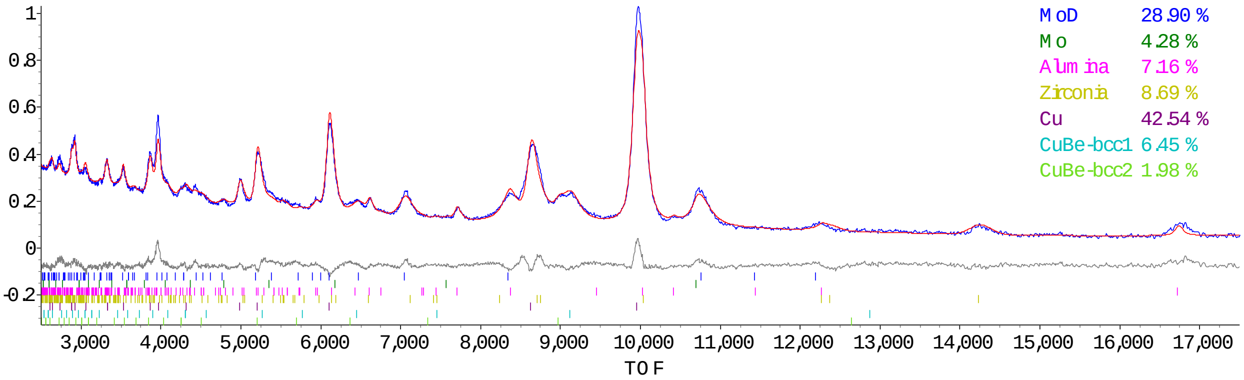

Figure S12: Rietveld refinement results of neutron powder diffraction experiments for 6.2 GPa. The y-axis is the relative intensities. On the PEARL instrument, d-spacing $\approx$ TOF/4842.

References

(S1) Feng, X.; Zhang, J.; Liu, H.; Iitaka, T.; Yin, K.; Wang, H. High Pressure Polyhydrides of Molybdenum: A First-principles Study. *Solid State Commun.* **2016**, *239*, 14–19.

(S2) Coelho, A. A. Topas V6.0. 2015; Brisbane, Australia.

(S3) Klotz, S.; Casula, M.; Komatsu, K.; Machida, S.; Hattori, T. High-Pressure Structure and Electronic Properties of YbD_2 to 34 GPa. *Phys. Rev. B* **2019**, *100*, 020101.
